# Inorganic Nitrogen Form Determines Nutrient Allocation and Metabolic Responses in Maritime Pine Seedlings

**DOI:** 10.3390/plants9040481

**Published:** 2020-04-09

**Authors:** Francisco Ortigosa, José Miguel Valderrama-Martín, José Alberto Urbano-Gámez, María Luisa García-Martín, Concepción Ávila, Francisco M. Cánovas, Rafael A. Cañas

**Affiliations:** 1 Grupo de Biología Molecular y Biotecnología, Departamento de Biología Molecular y Bioquímica, Universidad de Málaga, Campus Universitario de Teatinos, 29071 Málaga, Spain; fortigosa@uma.es (F.O.); jmvalderrama@uma.es (J.M.V.-M.); alburb@uma.es (J.A.U.-G.); cavila@uma.es (C.Á.); canovas@uma.es (F.M.C.); 2 BIONAND, Centro Andaluz de Nanomedicina y Biotecnología, Junta de Andalucía, Universidad de Málaga, 29590 Málaga, Spain; mlgarcia@bionand.es

**Keywords:** *Pinus pinaster*, nutrition, nitrate, ammonium, nitrogen use efficiency (NUE)

## Abstract

Nitrate and ammonium are the main forms of inorganic nitrogen available to plants. The present study aimed to investigate the metabolic changes caused by ammonium and nitrate nutrition in maritime pine (*Pinus pinaster* Ait.). Seedlings were grown with five solutions containing different proportions of nitrate and ammonium. Their nitrogen status was characterized through analyses of their biomass, different biochemical and molecular markers as well as a metabolite profile using ^1^H-NMR. Ammonium-fed seedlings exhibited higher biomass than nitrate-fed-seedlings. Nitrate mainly accumulated in the stem and ammonium in the roots. Needles of ammonium-fed seedlings had higher nitrogen and amino acid contents but lower levels of enzyme activities related to nitrogen metabolism. Higher amounts of soluble sugars and L-arginine were found in the roots of ammonium-fed seedlings. In contrast, L-asparagine accumulated in the roots of nitrate-fed seedlings. The differences in the allocation of nitrate and ammonium may function as metabolic buffers to prevent interference with the metabolism of photosynthetic organs. The metabolite profiles observed in the roots suggest problems with carbon and nitrogen assimilation in nitrate-supplied seedlings. Taken together, this new knowledge contributes not only to a better understanding of nitrogen metabolism but also to improving aspects of applied mineral nutrition for conifers.

## 1. Introduction

Nitrogen (N) is an essential element for life because it is a main constituent of biomolecules such as nucleic acids, proteins, chlorophylls, and hormones [1]. For plants, N is the main limiting nutrient due to the high amount that is needed to maintain sustained growth and its low availability in soil [2]. Although molecular dinitrogen is highly abundant in the atmosphere, it is not directly available to plants because it can only be assimilated by plant species in symbiosis with diazotrophic bacteria [2]. In the soil, there are different organic and inorganic forms of N that can be incorporated by plants. Nevertheless, the amount and proportions of these N molecules change depending on the climate and soil conditions as well as biological competition such as the decrease of soil nitrification caused by secondary metabolites from plant root exudates that inhibit the growth of nitrifying microorganisms [3].

The main forms of inorganic N that are available to plants in soil are ammonium and nitrate [4]. Their relative abundances in the soil have an important relationship because of the nitrification performed by microorganisms in the rhizosphere [5]. Ammonium is the initial substrate of the nitrification process, which is carried out under aerobic conditions and depends on temperature and pH [6]. Overall, plants tolerate or prefer different inorganic N forms depending on the soil in which they are grown. Most crops are adapted to temperate climates, so they grow well with N in the form of nitrate. Due to the economic importance of this fact, nitrate plant nutrition has been widely studied and is quite well understood [7,8,9]. The strong dependence of crop yield on N supply causes large amounts of N fertilizers to be applied to agricultural soils. This is economically and environmentally costly because it increases production costs and promotes environmental problems [8]. However, plants that are adapted to soils with low nitrification rates, such as rice or conifers, prefer or tolerate ammonium nutrition [10]. 

The assimilation of N into organic molecules always requires that N be in the form of ammonium. Thus, nitrate is reduced by nitrate reductase (NR, EC 1.7.1.1) using reduced nicotinamide adenine dinucleotide (NADH) and producing nitrite in the cytosol. Nitrite is toxic and quickly reduced in plastids by nitrite reductase (NiR, EC 1.7.2.1) using six molecules of reduced ferredoxin to produce ammonium. Nitrate reduction is a highly energy-consuming process that, depending on the plant species, takes place in the shoots or in the roots. The energy for the process comes from photosynthesis and is mainly expended in the NiR reaction. NiR employs ferredoxin that is directly reduced by the photosynthetic electron transport chain in photosynthetic tissues or is indirectly reduced by NADPH (reduced nicotinamide adenine dinucleotide phosphate): ferredoxin oxidoreductase (EC 1.18.1.2) in the amyloplasts of non-photosynthetic tissues using NADPH obtained from the photoassimilates that are transported from shoots to roots [1]. Ammonium is assimilated by the glutamine synthetase (GS, EC 6.3.1.2) /glutamate synthase (GOGAT, NADH-dependent EC 1.4.1.14; ferredoxin-dependent EC 1.4.7.1) cycle. GS produces glutamine from glutamate and ammonium expending one molecule of adenosine triphosphate (ATP). Glutamine is used by GOGAT with 2-oxoglutarate to produce two molecules of glutamate using the reduction power of NADH or ferredoxin depending on the enzyme isoform. One glutamate molecule feeds the cycle and the other is the net product of the cycle. Fd-GOGAT is mainly expressed in photosynthetic tissues, while NADH-GOGAT is more highly expressed in non-photosynthetic tissues [11]. In angiosperms and ginkgo, there is usually one gene encoding GS localized in the chloroplasts, GS2, and several genes encoding cytosolic isoforms, called GS1 [12]. GS2 and Fd-GOGAT act in the photosynthetic tissues and have important roles in the re-assimilation of ammonium released during photorespiration and the assimilation of ammonium from nitrate reduction [13]. GS1 and NADH-GOGAT manage the assimilation of N in roots and the reallocation of N in different metabolic and physiological processes such as senescence, fruit filling or stress responses [12,14,15]. All N compounds in plants are derived from the glutamine and glutamate produced in this cycle mainly through aminotransferase reactions [1]. 

Nitrate can be stored in plant vacuoles until its use without causing problems [16]. Nitrate is preferentially accumulated in different organs depending on the species and is remobilized from vacuoles to be reduced under adequate light conditions when photosynthesis is active because nitrate reduction is coupled to photosynthetic energy production [17]. However, excessive amounts of ammonium are toxic for most plant species even if large amounts of ammonium can be stored in the vacuoles [18]. Among the symptoms caused by ammonium excess there are chlorosis, growth suppression, yield depression, declines in cations such as potassium, calcium, and magnesium, increases in amino acids, and decreases in dicarboxylic acids such as malic acid, etc. [10]. 

Conifers are trees with long lifespans and life cycles, most of which have perennial leaves. Conifers cover vast areas of planet Earth. This confers extraordinary ecological importance to these plants because their forests are widely extended ecosystems, mainly located in the Northern Hemisphere [19]. Additionally, they represent a remarkable source of raw materials, such as wood or resin, for human use. Sustainable forest management is essential for obtaining adequate yields of these biomaterials while also conserving these ecosystems. As for all other plants, N is essential for conifer growth [20]. Most conifers prefer ammonium as the main inorganic N form for their growth [10,21,22]. This preference could be linked to the photorespiration process. It has been observed that pine saplings fed with nitrate as their sole N source grew slowly under high atmospheric CO_2_ concentrations and showed low photorespiration rates under normal or low atmospheric CO_2_ concentrations [23]. Interestingly, conifers have no GS2; rather, they have a cytosolic isoform, GS1a, in their photosynthetic tissues that has roles similar to those of GS2, including re-assimilation of ammonium released from photorespiration [14,24].

Maritime pine (*Pinus pinaster* Ait.) is a conifer tree from the southwestern Mediterranean region with great environmental and economic importance in France, Portugal, and Spain. However, it is also cultured and utilized in other regions. In some cases, it has become an invasive species, particularly in South Africa [25]. This species grows better with ammonium than with nitrate nutrition [20]. Despite its high phenotypic plasticity and high tolerance to abiotic stresses [26,27], this tree is adapted to live in temperate regions where conditions favor soil nitrification, at least during the warm seasons. Additionally, maritime pine has a complete set of transporters related to nitrate transport, including at least 40 members of the Nitrate Transporter 1/Peptide Transporter family (NPF) and 2 members of the Nitrate Transporter 2 family (NRT2), and an expanded family of nitrate transport regulators, 6 NRT3 [28]. The characterization of the response to inorganic nitrogen nutrition is of paramount importance to understand the development of plants, including trees, which have long life cycles. The aim of the present work is to determine the metabolic changes that the inorganic N form, nitrate or ammonium, causes in maritime pine seedlings. The main goals are to determine the differences in the incorporation rates of two inorganic nitrogen forms (nitrate/ammonium) and to analyze the effects that both nitrogen forms have on the growth and metabolism of pine seedlings, providing information that may serve as a precedent for future works on nitrogen nutrition in maritime pine and conifers.

## 2. Results

### 2.1. Biomass Accumulation and Seedling N Content in Response to Ammonium and Nitrate Supply 

**Figure 1:**
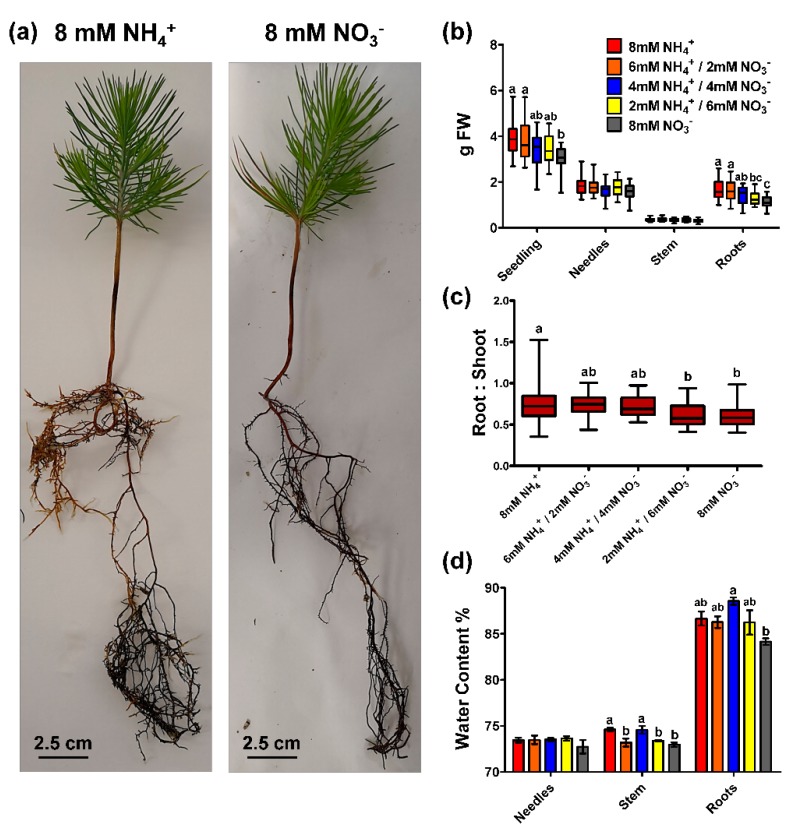
Biomass accumulation, root:shoot ratio, and water content in the different organs (needles, stem, and roots) of pine seedlings under the nutrient treatments. (**a**) Photographs of two seedlings after treatment with 8 mM ammonium and 8 mM nitrate; (**b**) Biomass accumulation in the whole seedling and the different organs (needles, stem, and roots); (**c**) Root:shoot ratio; (**d**) Water content. Red columns correspond to 8 mM NH_4_^+^ supply; orange columns correspond to 6 mM NH_4_^+^/2 mM NO_3_^−^ supply; blue columns correspond to 4 mM NH_4_^+^/4 mM NO_3_^−^ supply; yellow columns correspond to 2 mM NH_4_^+^/6 mM NO_3_^−^ supply; grey columns correspond to 8 mM NO_3_^−^ supply. Significant differences were determined with a one-way ANOVA for each organ or entire seedling. Letters above the columns show significant differences based on a Newman-Keuls post-hoc test (*p* < 0.05). Boxplots show minimum, maximum, and median values with *n* = 6 for biomass and root:shoot ratio. Error bars show SE with *n* = 3. FW corresponds to fresh weight.

The main goal of the present work was to evaluate the effect of the inorganic N form on maritime pine seedlings. Five different nutritional conditions were tested (Figure 1), all with a total N concentration of 8 mM but with different proportions of ammonium and nitrate (8/0 mM; 6/2 mM; 4/4 mM; 2/6 mM; and 0/8 mM, respectively). The first and most obvious effect of the different N sources was the differential biomass accumulation among seedlings (Figure 1a,b). Biomass decreased with the increase in nitrate content in the supplied solution, which was statistically significant in the case of seedlings fed with 8 mM nitrate. However, this effect was mainly caused by the root biomass where there were significant differences among seedlings. No differences among treatments were found in needle and stem biomass. This caused the root:shoot ratios to be significant higher in the seedlings supplied with more ammonium (Figure 1c). The water content was clearly higher in the roots than in the rest of the organs (Figure 1d). There were some differences in the water content between treatments in stem and roots with a slight tendency of a lower water content in the seedlings supplied only with nitrate.

**Figure 2:**
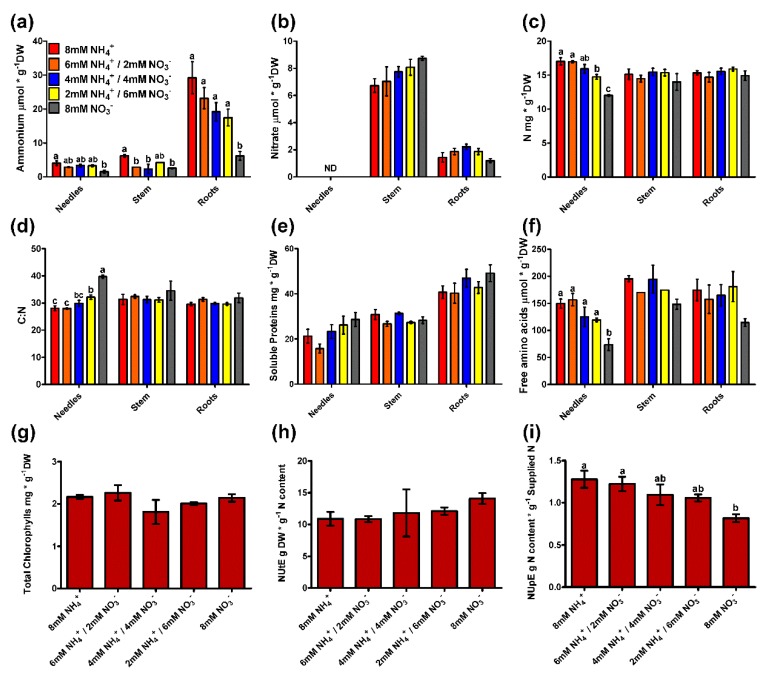
Profiles of plant N status markers in different organs (needles, stem, and roots) of pine seedlings under the nutrient treatments. (**a**) Ammonium content; (**b**) Nitrate content; (**c**) N content; (**d**) C:N ratio; (**e**) Soluble protein content; (**f**) Total free amino acid content; (**g**) Total chlorophyll content in pine seedling needles; (**h**) Nitrogen utilization efficiency (NUtE) in the seedlings under the different treatments; (**i**) Nitrogen uptake efficiency (NUpE) in the seedlings under the different treatments. Red columns correspond to 8 mM NH_4_^+^ supply; orange columns correspond to 6 mM NH_4_^+^/2 mM NO_3_^−^ supply; blue columns correspond to 4 mM NH_4_^+^/4 mM NO_3_^−^ supply; yellow columns correspond to 2 mM NH_4_^+^/6 mM NO_3_^−^ supply; grey columns correspond to 8 mM NO_3_^−^ supply. Significant differences were determined with a one-way ANOVA for each organ or entire seedling. Letters above the columns show significant differences based on a Newman-Keuls post-hoc test (*p* < 0.05). Error bars show SE with *n* = 3.

Ammonium mainly accumulated in the roots of the pine seedlings (Figure 2a). There were significant differences between ammonium-fed and nitrate-fed seedlings in every organ; these differences were most evident in the roots where there was 3 times more ammonium in the seedlings fed only ammonium than in those fed only nitrate. When the ammonium and nitrate contents were compared, the ammonium levels were higher in almost every case except in the stems of plants supplied with more nitrate (Figure 2a,b). The differences were more evident in the roots where the ammonium content was between 30–10 µmol g^−1^ dry weight (DW) and the nitrate content was approximately 1–2 µmol g^−1^ DW. There were no significant differences in the nitrate content among treatments, although there was a tendency for seedlings supplied with higher amounts of nitrate to accumulate nitrate in their stems (Figure 2b). However, there was an evident partitioning of nitrate accumulation between organs, with nitrate accumulation being higher in stems than in roots (nearly four times higher) and not being detected in needles. The form of inorganic N did not have an evident effect on the N content in the different organs (Figure 2c). The only significant differences were that the N content in the needles was higher in the seedlings supplied with higher amounts of ammonium (Figure 2c). In the rest of the organs, no significant differences were observed, though the N content was generally lower in the seedlings fed only nitrate. The patterns above resulted in significant differences in the carbon:nitrogen (C:N) ratios in the needles, which were higher in the seedlings supplied with more nitrate. There were no significant differences in the C:N ratios in the rest of the organs, although the ratio was slightly higher in the seedlings fed only nitrate (Figure 2d). This is because there were no significant differences in the carbon (C) content among the seedlings. The nitrogen use efficiency (NUE) reflects differences among treatments in N uptake (Figure 2i) but not in N utilization (Figure 2h). The nitrogen utilization efficiency (NUtE) was slightly higher in seedlings supplied with more nitrate, while the nitrogen uptake efficiency (NUpE) tended to be higher in seedlings fed with more ammonium, with significant differences from the seedlings only supplied with nitrate.

Additionally, different indicators of the N status that are usually considered N sinks were measured. Soluble proteins and total chlorophylls are two good markers of the N content [2]. In the present work, there were no significant differences in these two parameters among the nutrient treatments (Figure 2g,e). However, soluble proteins tended to accumulate more in needle and root samples from seedlings fed with higher amounts of nitrate (Figure 2e). An additional analyzed N marker was the free amino acid content (Figure 2f). In the needles, significantly lower levels of amino acids were detected when the plants were supplied only with nitrate than when the plants were grown under the other conditions. No significant differences were observed in the amino acid levels of the stems and roots among the different treatments, although there was a tendency for plants supplied only with nitrate to accumulate lower levels of amino acids.

### 2.2. ^15^N-labeled Ammonium and Nitrate Uptake

**Figure 3:**
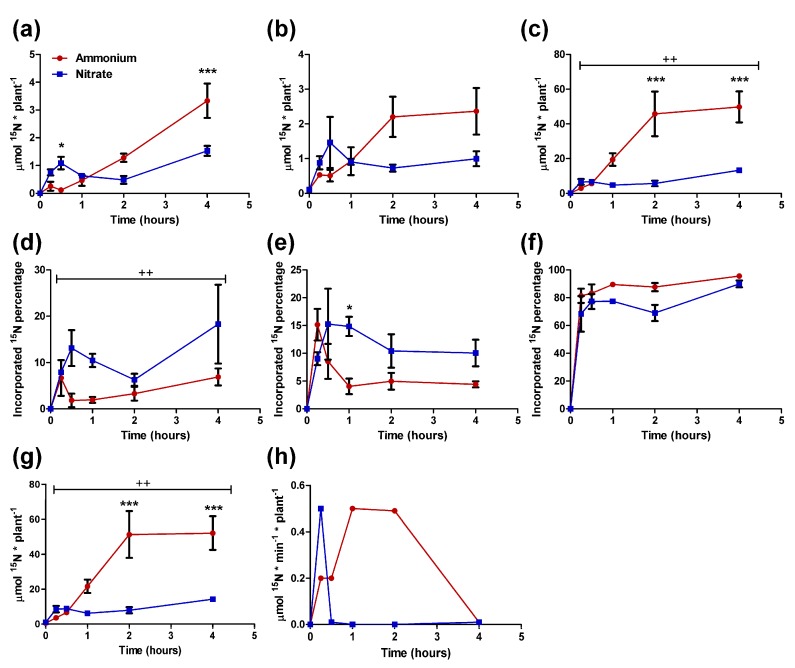
^15^N incorporation in different organs (needles, stem, and roots) of pine seedlings under the two nutrient treatments. The red line corresponds to 7.5 mM ^15^N-labeled ammonium. The blue line corresponds to 7.5 mM ^15^N-labeled nitrate. ^15^N amount in needles (**a**); stems (**b**); roots (**c**); and the whole seedling (**g**). Percentage of ^15^N contained in needles (**d**); stems (**e**); and roots (**f**) with respect to the amount in the whole seedling. ^15^N Incorporation rate in the seedling (**h**). Differences between treatments were determined with a two-way ANOVA. Significant differences are indicated with crosses (++ at *p* < 0.01). Differences between treatments in each individual time point were determined with a Bonferroni post-hoc test. Significant differences are indicated with asterisks on top of the columns: * at *p* < 0.05; ** at *p* < 0.01, *** at *p* < 0.001. Error bars show SE with *n* = 3.

Considering the results for NUpE, the N uptake was analyzed with ^15^N-labeled ammonium and nitrate (Figure 3). One-month-old seedlings were fed with 7.5 mM of ^15^N-labeled ammonium or nitrate. The N incorporation was determined through the measurement of the ^15^N content in the different organs. As expected, ^15^N accumulation was much higher in the roots than in the other organs (Figure 3a–c). In every organ, the ^15^N incorporation during the first 30 min was higher in nitrate-fed seedlings than in ammonium-fed seedlings. From the first hour, there was an inversion of this trend, with higher ^15^N incorporation in the ammonium seedlings. This was due to an increase in ^15^N accumulation from 30 min to 2 h, although the amount in stems and roots was stable from 2 h to the end of the experiment in the ammonium-fed seedlings, and the ^15^N content stabilized in seedlings fed with nitrate (Figure 3a–c,g). This was statistically significant in roots and in whole seedlings, although the profiles were similar for every organ. The ^15^N incorporation rate was very high for the first 15 min in nitrate-fed seedlings, followed by an extreme decrease in the ^15^N incorporation until the end of the experiment (Figure 3h). For the ammonium condition, the incorporation rate increased from 15 min to one hour, maintaining its value until 2 h and decreasing to minimum levels at the end of the assay (Figure 3h). The distribution of ^15^N was analyzed using the percentage of ^15^N in each organ with respect to the total ^15^N in the seedlings (Figure 3d–f). In the needles and stems, the percentages of ^15^N were higher in nitrate seedlings than in ammonium seedlings from 15 and 30 min to the final point, although the difference was only significant in needles. However, these percentages were inverse in the roots, being higher in ammonium-fed seedlings than in nitrate-fed seedlings at every time point.

### 2.3. Enzyme Activity and Gene Expression Profiles 

**Figure 4:**
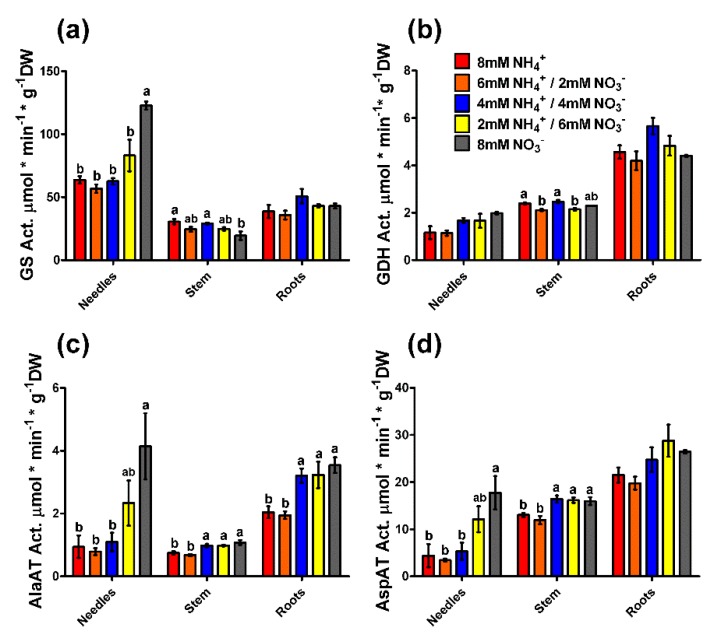
Enzyme activity in the different organs (needles, stem, and roots) of pine seedlings under the nutrient treatments. (**a**) Glutamine synthetase (GS, EC 6.3.1.2) activity; (**b**) Glutamate dehydrogenase (GDH, EC 1.4.1.3) activity; (**c**) Alanine aminotransferase (AlaAT, EC 2.6.1.2) activity; (**d**) Aspartate aminotransferase (AspAT, EC 2.6.1.1) activity. Red columns correspond to 8 mM NH_4_^+^ supply; orange columns correspond to 6 mM NH_4_^+^/2 mM NO_3_^−^ supply; blue columns correspond to 4 mM NH_4_^+^/4 mM NO_3_^−^ supply; yellow columns correspond to 2 mM NH_4_^+^/6 mM NO_3_^−^ supply; grey columns correspond to 8 mM NO_3_^−^ supply. Significant differences were determined with a one-way ANOVA for each organ or entire seedling. Letters above the columns show significant differences based on a Newman-Keuls post-hoc test (*p* < 0.05). Error bars show SE with *n* = 3.

Some of the enzymatic activities and the expression levels of genes coding for the main actors in N metabolism were measured in the present work (Figure 4). GS activity was higher in needles than in stems and roots (by 2–3 times) (Figure 4a). In needles, GS activity was significantly higher only in the nitrate-fed seedlings and tended to increase with the nitrate supply. The opposite effect was observed in the stem, with a significant decrease in GS activity in the 8 mM nitrate treatment and a tendency to decrease with the nitrate supply. The different treatments did not have a clear effect on glutamate dehydrogenase (GDH) activity, although in the stems, there were significant differences (Figure 4b). The GDH activity increased from the top to the bottom of the seedlings. There was a slight and not statistically significant increase in GDH activity in the needles in parallel to the increase in the nitrate supply. Alanine and aspartate aminotransferase (AspAT and AlaAT, respectively) activities increased from the needles to the roots and were, in general, significantly higher under the conditions with more nitrate, except for the AspAT activity in the roots where no significant differences were observed (Figure 4c,d).

**Figure 5:**
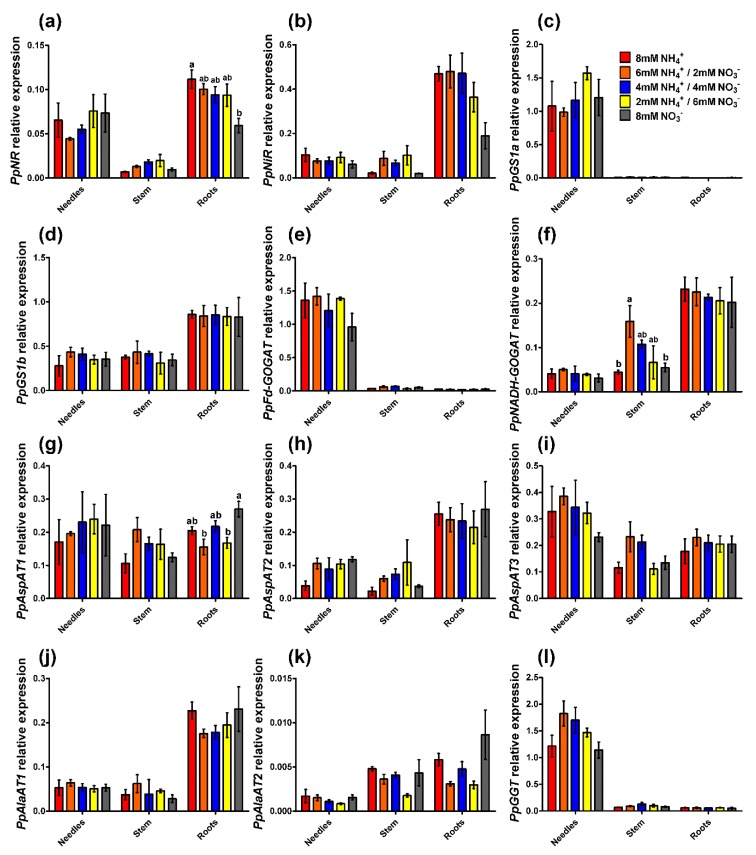
Gene expression profiles in the different organs (needles, stem, and roots) of pine seedlings under the nutrient treatments. (**a**) Nitrate reductase (*PpNR*); (**b**) Nitrite reductase (*PpNiR*); (**c**) Glutamine synthetase 1a (*PpGS1a*); (**d**) Glutamine synthetase 1b (*PpGS1b*); (**e**) Ferredoxin dependent glutamate synthase (*PpFd-GOGAT*); (**f**) NADH-dependent glutamate synthase (*PpNADH-GOGAT*); (**g**) Aspartate aminotransferase 1 (*PpAspAT1*); (**h**) Aspartate aminotransferase 2 (*PpAspAT2*); (**i**) Aspartate aminotransferase 3 (*PpAspAT3*); (**j**) Alanine aminotransferase 1 (*PpAlaAT1*); (**k**) Alanine aminotransferase 2 (*PpAlaAT2*); (**l**) Glyoxylate-glutamate aminotransferase (*PpGGT*). Red columns correspond to 8 mM NH_4_^+^ supply; orange columns correspond to 6 mM NH_4_^+^/2 mM NO_3_^−^ supply; blue columns correspond to 4 mM NH_4_^+^/4 mM NO_3_^-^ supply; yellow columns correspond to 2 mM NH_4_^+^/6 mM NO_3_^−^ supply; grey columns correspond to 8 mM NO_3_^−^ supply. Significant differences were determined with a one-way ANOVA for each organ or entire seedling. Letters above the columns show significant differences based on a Newman-Keuls post-hoc test (*p* < 0.05). Error bars show SE with *n* = 3.

The expression of the gene encoding nitrate reductase (*PpNR*) was mainly observed in the needles and roots and was very low in the stems (Figure 5a). In roots, *PpNR* expression was significantly lower in plants under 8 mM nitrate supply, with the highest expression in the roots of seedlings under 8 mM ammonium. The expression profile of the gene encoding nitrite reductase (*PpNiR*) was similar to that of *PpNR*, with two exceptions; the expression in needles was low in comparison to that in roots, and the differences in the roots between treatments were not significant, although the profile was the same as that for *PpNR* expression (Figure 5b). The expression of both *GS* genes, *PpGS1a* and *PpGS1b*, had no significant differences among treatments (Figure 5c,d). *PpGS1a* was mainly expressed in the needles, while *PpGS1b* was expressed in all the organs, especially in the roots, where the levels were twice those in the needles and stems. The expression profile of the gene encoding ferredoxin-dependent glutamate synthase (*PpFd-GOGAT*) was similar to that of *PpGS1a*; it was expressed mainly in the needles and showed only a slight tendency to decrease in seedlings supplied with nitrate (Figure 5e). However, the transcript levels for the NADH-dependent enzyme (*PpNADH-GOGAT*) among organs were similar to those observed for *PpGS1b,* with higher levels in the roots than in the other organs (Figure 5f). Additionally, the expression of *PpNADH-GOGAT* in the stems changed significantly among the treatments; the highest expression was observed in the seedlings with 6 mM ammonium/2 mM nitrate. Lower expression was observed in the seedlings with 8 mM ammonium and 8 mM nitrate with a tendency to diminish its expression with the increase in the nitrate supply. Furthermore, the expression levels of genes encoding enzymes involved in the first use of assimilated N in the form of glutamate were analyzed, i.e., aspartate aminotransferase (*PpAspAT*), alanine aminotransferase (*PpAlaAT*), and glyoxylate-glutamate aminotransferase (*PpGGT*). For all three pine *PpAspAT* genes, there was no clear expression profile related to the nutritional treatments, although in most of the cases, the expression level in 8 mM ammonium-fed seedlings was the lowest (Figure 5g–i). Only *PpAspAT1* expression in the roots exhibited some significant differences, being higher in the 8 mM nitrate treatment than in the 6 mM ammonium/2 mM nitrate and 2 mM ammonium/6 mM nitrate treatments. *PpAspAT1* encodes a cytosolic protein, and the expression levels among organs were similar. However, the product of *PpAspAT2* has a predicted mitochondrial location, while that of *PpAspAT3* has a putative chloroplastic location. *PpAspAT2* was most highly expressed in roots (Figure 5h), and *PpAspAT3* was most highly expressed in needles (Figure 5i). *PpAlaAT1*, which produces a mitochondrial localized protein, had a moderate expression that was higher in the roots (Figure 5j). Nevertheless, *PpAlaAT2* expression was extremely low and almost residual (Figure 5k). As expected, the expression of the *PpGGT* gene was very high in the needles and did not show significant differences among treatments despite its lower expression in the 8 mM ammonium and 8 mM nitrate seedlings in comparison to that for the rest of the conditions, especially in the 6 mM ammonium/2 mM nitrate treatment (Figure 5l). 

### 2.4. Metabolite Profiling 

**Figure 6:**
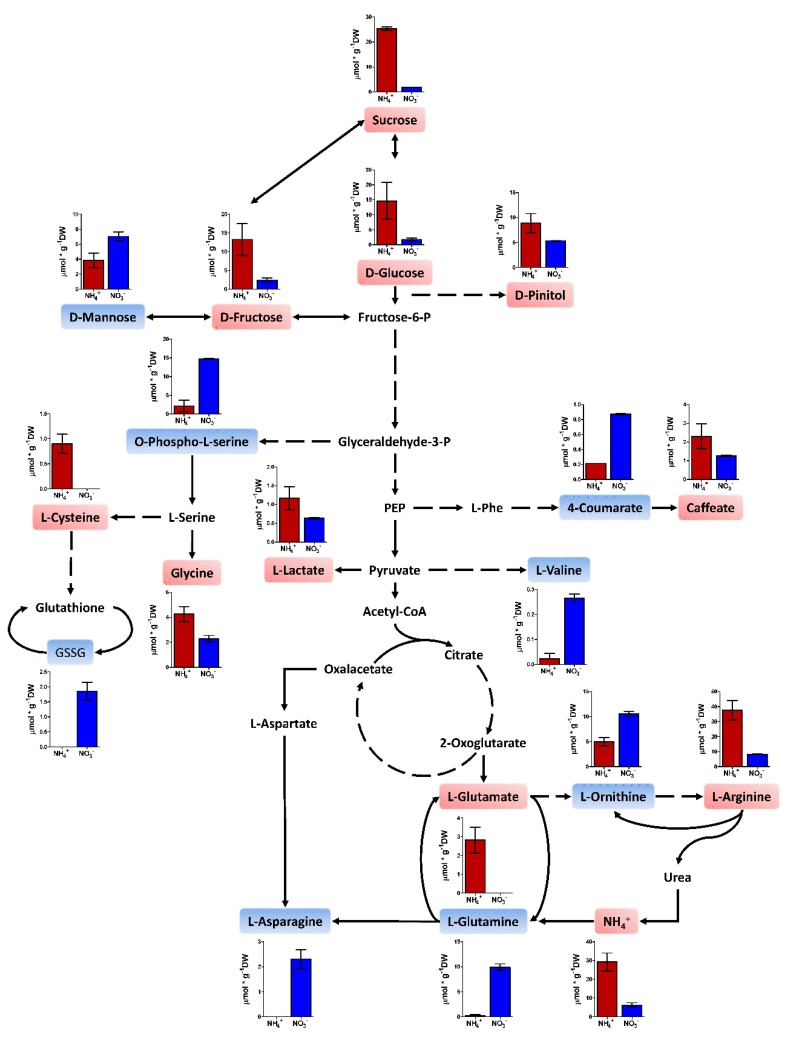
Main significant metabolites in the roots of seedlings fed 8 mM ammonium or 8 mM nitrate. Red columns correspond to 8 mM NH_4_^+^ supply; blue columns correspond to 8 mM NO_3_^−^ supply. Metabolites highlighted in red were significantly more accumulated in the roots of seedlings fed 8 mM ammonium. Metabolites highlighted in blue were significantly more accumulated in the roots of seedlings fed 8 mM nitrate. GSSG: Oxidized glutathione; PEP: Phosphoenolpyruvate; L-Phe: L-Phenylalanine. Significant differences were determined with a *t*-test (FDR < 0.05). Error bars show SE with *n* = 3.

The observed changes in the N status, enzyme activities, and gene expression should be reflected in equivalent changes in the metabolome. Due to this hypothesis, moderate profiling of polar metabolites was performed using proton nuclear magnetic resonance (^1^H-NMR). In the end, 69 metabolites were determined, including different sugars, amino acids, and some secondary metabolites. The results are presented in Table S1. As expected, a heatmap analysis of the metabolite profile shows that samples are grouped by organ (Figure S1). L-arginine was the main metabolite affected by the nutritional treatments in a global ANOVA (Figure S2). When the extreme treatments (8 mM NH_4_^+^ and 8 mM NO_3_^−^) were analyzed in each organ in an independent manner, significant differences (25 metabolites) between treatments were found in the roots (Table S1 and Figure 6). Notably, some of the main carbohydrates in pines such as sucrose, D-fructose, D-glucose, and D-pinitol were present at higher levels in ammonium-fed seedlings than in nitrate-fed plants. The same effect was found for several amino acids such as L-glutamate, L-arginine, L-cysteine, and glycine. However, O-phospho-L-serine, L-valine, L-ornithine, L-glutamine, and L-asparagine were present in higher amounts in the roots of nitrate-fed seedlings. D-mannose, 4-coumarate, and oxidized glutathione contents were also higher in roots of nitrate-fed seedlings.

## 3. Discussion

The preference of conifers for different inorganic N forms has been previously discussed in different works [21,22,29]. Depending on the species habitat, including climatic and soil conditions, conifers show preferences for the uptake and utilization of ammonium or nitrate [22]. Most of the conifers are tolerant to ammonium [20,30]. In the case of *P. pinaster*, its growth is higher with ammonium than with nitrate [21], although it is a conifer species with extensive families of nitrate transporters (NPF and NRT2) and transport regulators (NRT3) [28] and also lives in the Western Mediterranean region where climate conditions can promote high nitrification rates in the soil. In this context, the goal of the present work was to identify the main changes caused at the metabolic level by ammonium or nitrate supply in maritime pine seedlings. 

The results included in this study considerably expand the knowledge of N nutrition in maritime pine provided by previous reports. The seedlings with a higher ammonium supply grew better (Figure 1), but the whole plants also accumulated more N through higher N uptake efficiency (NUpE) (Figure 2). In fact, the ^15^N labeling experiment supported this observation; the ammonium uptake in seedlings was higher, and there was a high incorporation rate (at the µmolar level) during most part of the experiment (Figure 3). Interestingly, the differential biomass accumulation took place mainly in the roots, causing an increase in the root:shoot ratio in ammonium-fed seedlings. The application of high amounts of ammonium usually inhibits root growth and decreases the root:shoot ratio in plants [10]. In maritime pine seedlings, it seems that root growth increases following ammonium application, or at least, the growth inhibition is lower than that in seedlings fed with nitrate (Figure 1). In conifers, the root growth and the root:shoot ratio vary depending on the N source, which can include organic compounds such as L-arginine, which favors root development and a high root:shoot ratio [31]. In fact, this is considered a good trait for the field establishment of conifer seedlings [31,32]. Thus, the above results support the preference of maritime pine for ammonium as an inorganic N form over nitrate and suggest that the ammonium preference promotes beneficial root development. In this context, roots were the only organ with significant differences in metabolite content among treatments in the present study (Figure 6, Table S1). This is significant considering that roots seem to be the organ where primary N assimilation occurs in pine (Figure 5), despite the resolution of the analytical procedure being only in the mM range (400 MHz NMR spectrometer).

The N content clearly increased in the seedlings supplied with ammonium that had higher N uptake efficiency (NUpE) (Figure 2). However, changes in the N content occurred mainly in the needles. Considering the N partitioning, the free amino acid content seems to be the main factor responsible for this effect, with slight participation from the ammonium content (Figure 2). The metabolite profile in roots also suggests that ammonium-fed plants had a better N status (Figure 6, Table S1). Certain amino acids are good markers of a healthy N status, such as L-glutamate, L-cysteine, and L-arginine. L-glutamate is the net product of the GS/GOGAT cycle, which is the pathway mainly responsible for N assimilation [14]. L-cysteine is the final product in the sulfur assimilation pathway, but its biosynthesis depends on the availability of assimilated N [33]. Furthermore, L-cysteine acts as a precursor for antioxidants and defense compounds [34] and is related to the transcriptional response of ammonium nutrition in plants that have a greater tolerance for ammonium [35]. L-arginine is synthesized from L-glutamate and is an amino acid with an important role as an N reserve in pine. This amino acid is very abundant in storage proteins and is an important sink for assimilated N surplus [20,36]. Additionally, the metabolite profile in the roots indicates that the availability of C for metabolic processes was reduced in nitrate-fed seedlings (Figure 6, Table S1). The levels of the main soluble sugars, such as sucrose, D-fructose, and D-glucose, were extremely low in nitrate-fed seedlings in comparison to those in ammonium-fed seedlings. In fact, some C sinks such as D-pinitol or caffeic acid were also more accumulated in the ammonium-feed seedlings. This correlates well with the accumulation of L-asparagine, L-glutamine, and L-ornithine in the nitrate-fed plants. L-asparagine is an amino acid that is synthesized from L-glutamine and L-aspartate and is employed as a temporal N reserve when C is depleted [37]. Similarly, the accumulation of L-ornithine could suggest the active catabolism of L-arginine to mobilize the stored N and produce L-glutamate [38]. Interestingly, in the roots of the ammonium-fed seedlings, a greater amount of choline (4 times) was observed than in the roots of nitrate-fed plants (Table S1). Choline is the precursor of glycinebetaine in most living organisms and it is well known to play a role in osmotic stress [39], which could be related to ammonium levels since a higher water content was observed in the ammonium-fed seedlings compared to nitrate-fed plants (Figure 1d). Furthermore, glycinebetaine plays a role in oxidative stress responses by enhancing antioxidative responses [39,40] which could be linked to the transcriptomic response to ammonium [41]. 

Interestingly, the main accumulation of nitrate and ammonium was not in needles. This suggests that the changes in N content in the needles were related to metabolic processes associated with N management (assimilation and recycling). Additionally, the partitioning of ammonium and nitrate within the seedlings was different. The seedlings accumulated the most ammonium in the roots, avoiding major increases in its concentration in aerial organs. In plants, primary ammonium assimilation generally occurs in the roots [42]. Pine plants may subtly regulate and buffer the ammonium content in their organs via primary assimilation in the roots and the accumulation of the ammonium excess in the same organ, probably in the vacuoles [18,43]. This regulatory mechanism can prevent problems derived from the high levels of ammonium released during photorespiration in the photosynthetic tissues or during lignification that occurs mainly in the stem [14]. Additionally, free ammonium levels were higher in seedlings fed ammonium, mainly in the roots. However, the differences in nitrate content between the different treatments were not very large (not statistically significant), and the levels of nitrate accumulation were several times lower than the ammonium accumulation in the same organs. This fact and the total N content of the seedlings suggest that ammonium uptake is less restricted than nitrate uptake in maritime pine. The nitrate incorporation rate was only higher than the ammonium incorporation rate during the first 15 min, suggesting precise regulation by nitrate transporters (Figure 3). This low nitrate uptake rate has also been observed in white spruce, and the authors proposed that nitrate uptake systems are atrophied in plants that prefer ammonium [4,44]. However, maritime pine possesses a complete set of nitrate transporters and even an expanded gene family that encodes nitrate transport regulators (NRT3). In this context, it is tempting to speculate that maritime pine senses nitrate to be a toxic molecule. 

Although it is well known that plants prefer to accumulate nitrate over ammonium, which can produce cellular toxicity in several ways [10,42], pine is able to store more ammonium than nitrate at similar supply levels. Curiously, excess nitrate is mainly stored in the stem, an organ with a less important N assimilatory role than needles and roots, as suggested by the *PpNR* and *PpNiR* expression levels (Figure 5). This observation, along with the observed content of L-arginine in the stem (Figure S2) and the accumulation of L-asparagine in the seedling hypocotyl during the postgermination phase [38,45], suggests that the pine stem has a role as a store of N that accumulates not only vegetative storage proteins (VSPs) in the bark [46], but also free metabolites such as nitrate, L-arginine or L-asparagine. It is known that trees are able to transiently accumulate N in free amino acids [47]; in the future, it will be interesting to analyze the role of adult pine stems in the storage of N through the accumulation of small metabolites such as nitrate or free amino acids. 

Interestingly, nitrate did not accumulate in the needles (Figure 2), and the activity of enzymes involved in basal N metabolism increased in the needles with the nitrate supply (Figure 4). These results suggest a limited ability for nitrate assimilation in maritime pine, which may be related to the photorespiration pathway. Conifers lack a chloroplastic GS isoform (GS2) that is involved in the photoassimilation of nitrate and the reassimilation of ammonium released during photorespiration in angiosperm plants [14]. This could explain the increase in GS activity in the needles of nitrate-fed seedlings (Figure 4). In fact, a strong relationship between nitrate assimilation and photorespiration in pine has been observed in *Pinus taeda* saplings, which grew better with nitrate under low CO_2_ concentrations than under elevated CO_2_ concentrations; in contrast, CO_2_ concentration had no effect in the growth rate of ammonium-fed saplings [23]. However, it seems that needle metabolism is influenced by the form of available inorganic N. In this context, these effects appear to be regulated through the allocation of the inorganic N forms to the different organs and through their assimilation in the roots, as indicated by the expression levels of *PpNR* and *PpNiR* genes (Figure 5). Despite the expression of these genes, the ^15^N incorporation assay indicated that nitrate was relatively better transported from roots to stem and needles than ammonium (Figure 3d–f), at least when the nitrate incorporation rate was high. It is possible that nitrate assimilation into the needles negatively affects photosynthetic/photorespiration metabolism, inducing a negative feedback with nitrate transport in the roots. Nitrate uptake inhibition has been previously observed in different plants when enough nitrate is assimilated, but not in such a drastic manner [48].

Another interesting finding is the lack of correlation between enzyme activity (GS, AspAT, and AlaAT) and the expression of the genes coding for the enzymes that catalyze these reactions (Figure 3 and Figure 4). These findings suggest that the response to the N form must be regulated through a post-transcriptional (translational or post-translational) mechanism. This could involve changes in translation or in the proteolysis rates. A second mechanism has been proposed for the GS enzyme in mammals, wherein the increase in glutamine levels drives ubiquitination and proteasome degradation of the protein [49,50]. In plants, ubiquitination-dependent proteolysis also plays a role in N metabolism during plant adaptation to N starvation [51,52].

## 4. Materials and Methods 

### 4.1. Plant Material

Maritime pine seeds (*Pinus pinaster* Ait.) from Sierra Bermeja (Estepona, Spain) (ES20, Ident. – 11/12) were obtained from the *Área de Recursos Genéticos Forestales* of the Spanish *Ministerio de Agricultura, Pesca y Alimentación*. Pine seeds were imbibed in distilled water for 48 h under continuous aeration and germinated with vermiculite as an inert substrate. Pine seedlings were cultivated in a growth chamber with a 16/8 h light/dark photoperiod, light intensity of 125 μmol m^−2^ s^−1^, constant temperature of 26–27 °C, and 75%–80% relative humidity. Forty seedlings aged one month were randomly transplanted into forestall seedbeds for each experimental condition. The seedlings were grown in a greenhouse from February to April of 2019 at a mean temperature of 25 °C and 50/70% relative humidity with a 16/8-h photoperiod (*Instituto de Hortifruticultura Subtropical y Mediterránea*, IHSM *La Mayora* UMA-CSIC). Each group was irrigated twice per week with 40 mL of the corresponding N solution or 40 mL of distilled water. Five experimental conditions were tested: 8 mM NH_4_Cl; 6 mM NH_4_Cl–2 mM KNO_3_; 4 mM NH_4_Cl–4 mM KNO_3_; 2 mM NH_4_Cl–6 mM KNO_3_; 8 mM KNO_3_. After 60 days of treatment, seedlings were subdivided and harvested in three random groups. The seedlings were divided into three different sections (cotyledon, hypocotyl, and roots). Each section was weighed and immediately frozen in liquid N_2_. For the N uptake analyses, one-month-old seedlings grown in vermiculite and only irrigated with distilled water were used. They were separated into two groups. One group was fed with 7.5 mM of ^15^N-labelled ammonium and the second one with 7.5 mM of ^15^N-labelled nitrate. The plants were harvested at different times after nutrient application: 0, 15, 30, 60, 120, and 240 min. Each organ was isolated, weighed, and immediately frozen in liquid N_2_. Three biological replicates were taken. Each replicate consisted of a pool of five seedlings. All samples were stored at −80 °C until powdering with a mixer mill MM400 (Retsh, Haan, Germany) and further analyses were conducted.

### 4.2. Elemental Analysis and NUE Component Estimation

Ground powder (100 mg) of cotyledons, hypocotyls, and roots was dried at 70 °C for 48 h in an oven. Total C and N contents in different sections of pine seedlings were determined in triplicate by an elemental macro-analyzer Leco truSpec CHNS (Leco Corporation, St. Joseph, MI) at the Atomic Spectrometry Unit, University of Málaga. ^15^N determinations were performed by mass spectrometry using a Flash IRMS Elemental Analyzer (EA-IRMS), Delta V IRMS, Conflo IV Universal Interface (Thermo Scientific, MA, USA). The N content and biomass of the samples were used to calculate the nitrogen utilization efficiency (NUtE) and nitrogen uptake efficiency (NUpE) [53]. 

### 4.3. Free Amino Acids, Ammonium, Nitrate, and Nitrite Contents

Free amino acids and ammonium were extracted with 2% 5-sulfosalicylic acid (100 mg FW mL^−1^) [54]. Soluble amino acids were determined using the procedure described by Sun et al. [55]. Free ammonium was measured using the Berthelot reaction (phenol hypochlorite assay) [56]. Nitrate and nitrite measures were performed as described by García-Robledo et al. [57]. Nitrite was determined spectrophotometrically at 540 nm after a colorimetric reaction using Griess’ reactive (equal volumes of 60 mM sulfanilamide in 1.2 N HCl and 4 mM N-(1-naphtyl) ethylendiamine dihydrochloride) after 20 min incubation at room temperature. Nitrate was reduced to nitrite with 2% vanadium (III) chloride (VCl_3_) in 6N HCl, and the produced nitrite was measured as described above. Nitrite was measured prior the nitrate reduction with the aim of determining the basal nitrite present in the samples. Nitrite and nitrate contents were calculated based on a standard curve using commercial sodium nitrite from Sigma–Aldrich (MO, USA).

### 4.4. Soluble Protein, Enzyme Activity, and Chlorophyls Determinations

Soluble proteins were extracted using 100 mg of sample ground powder for stem and roots and 50 mg in the case of needles. The extraction was performed by adding 1 mL of extraction buffer (50 mM Tris-HCl pH 8, 1 mM EDTA, 10 mM MgCl_2_, 0.5 mM dithiothreitol (DTT), 20% (*w/v*) glycerol, 0.1% (*v/v*) Triton X-100, 1% (*w/v*) polyvinylpyrrolidone (PVP), and 1% (*w/v*) polyvinyl(poly)pyrrolidone (PVPP)) and 30 mg of fine sea sand. The resulting extract was centrifuged at 12,000 g for 30 min at 4 °C. The obtained supernatants were recovered and used for soluble protein determination through Bradford’s procedure using a commercial reagent (Protein Assay Dye Reagent; Bio-Rad, CA, USA) and bovine serum albumin as a standard [58].

Glutamine synthetase (GS, EC 6.3.1.2) activity was determined by the transferase assay following Cánovas et al. [59]. The final reaction volume was 150 µL. Reactions were incubated for 15 min at 37 °C with 10 s of agitation every minute; the reactions were stopped with 150 µL of STOP solution (10% FeCl_3_·6H_2_O in 0.2 N HCl, 24% trichloroacetic acid and 50% HCl) and centrifuged for 3 min at 3220 g. After centrifugation, 200 µL of the supernatant was recovered, and absorbance was measured at 540 nm in a microplate reader. For estimation of glutamate dehydrogenase (GDH, EC 1.4.1.2) activity, the NADH-GDH assay was used [60]. The reaction was developed in a final volume of 100 µL. Aspartate aminotransferase (AspAT, EC 2.6.1.1) and alanine aminotransferase (AlaAT, EC 2.6.1.2) activities were measured following Gibon et al. [61] in a final reaction volume of 100 µL.

Chlorophyll extraction was performed using 50 µL of protein extract mixed with 950 µL of 80% (*v/v*) acetone. Samples were incubated at 4 °C overnight. Resulting extracts were centrifuged at 13,500 g at 4 °C for 10 min, and the absorbance was measured at 664 and 647 nm. The chlorophyll content was calculated according to Lichtenthaler and Buschmann [62].

### 4.5. Metabolite Profiling

The metabolites for ^1^H-NMR analysis were extracted following the protocol previously described by Kruger et al. [63]. Two hundred milligrams of frozen powder were used for extractions. The ^1^H-NMR analyses were performed on a Bruker ASCEND™ 400 MHz NMR Spectrometer (Bionand, *Centro Andaluz de Nanomedicina y Biotecnología*, Málaga, Spain). The 1D-^1^H-NMR spectrum for each sample was obtained as previously described by Cañas et al. [64]. Quantitative analysis of the NMR spectra was performed using LCModel software (Linear Combination of Model Spectra) [65] and a previously generated reference metabolite spectral library [64]. The internal reference was an electronically generated signal, ERETIC (electronic reference to access in vivo concentrations) [66]. The metabolite amounts were determined at millimolar concentrations. 

The metabolite contents were analyzed with MetaboAnalyst 4.0 [67]. Data were normalized using the quantile method, then log transformation and mean centered. MetaboAnalyst 4.0 was used to construct a Heatmap and perform a t-test with the metabolite data.

### 4.6. RNA Extraction and Reverse-Transcription Quantitative PCR (RT-qPCR)

RNA was extracted as described by Canales *et al.* [68] from ground powder stored at −80 °C. A treatment with RQ1 RNase-Free DNase (Promega, Wis, USA) was applied to remove genomic DNA from the RNA samples. Total RNA quantification and purity were estimated using a NanoDrop ND-1000 spectrophotometer (Thermo Scientific, MA, USA), and RNA integrity was checked by agarose gel. Reverse transcription reactions were performed using iScrpt^TM^ Reverse Transcription Supermix (Bio-Rad, CA, USA) using 1 µg of total RNA. The qPCR reactions were carried out using 5 ng of cDNA and SsoFast™ EvaGreen^®^ Supermix (Bio-Rad, CA, USA) in a final volume of 10 µL. The reactions were developed on a C1000TM Thermal Cycler with a CFX384TM Touch Real-Time PCR Detection System (Bio-Rad, CA, USA) under the following conditions: 3 min at 95 °C (1 cycle), 1 s at 95 °C, and 5 s at 60 °C (50 cycles), with a melting curve from 60 °C to 95 °C. The raw fluorescence data from each reaction were fitted to the MAK2 model [69]. The initial target concentration (D0 parameter) was determined using the R package *qpcR* [70]. Expression data were normalized to two reference genes, *SKP1/ASK1* and *SLAP,* that were previously tested for RT-qPCR experiments in maritime pine [71]. For the qPCR analysis, three biological replicates and three technical replicates per sample were used. Primers used for qPCR are presented in Table S2.

### 4.7. Statistics

Biomass and root:shoot ratio results are presented in boxplots including minimum, maximum, and median values. For the rest of experiments, the mean values for three pools of plants with standard errors (SE = SD/√(n-1)) are presented. Statistical analyses were performed using Prism 5 (Graphpad, CA, USA) except for metabolite data that were analyzed using MetaboAnalyst 4.0 [67]. Differences among organs were not statistically analyzed. For each organ and whole seedling, nutritional differences were statistically analyzed to reduce problems with distribution and variance of data. In this line, one-way ANOVA was used for the analyses of all data except for ^15^N incorporation and metabolite profile assuming that data met ANOVA conditions. When one-way ANOVA was significant, a Newman-Keuls multiple comparison test was carried out. The ^15^N incorporation time experiment was analyzed using two-way ANOVA (mixed model) with a Bonferroni post-test determining significant differences in nutrition conditions between the global time experiment and each individual time point. Root metabolite profiles were analyzed using *t*-tests. In every case, significant differences were considered when *p* < 0.05 except for *t*-test analyses, where FDR < 0.05 was assumed. 

## 5. Conclusions

The results of the present work demonstrate that ammonium and nitrate nutrients behave differently from each other in pine, although their assimilation into organic molecules occurs through the same pathway, the GS/GOGAT cycle. Their chemical characteristics and the reduction of nitrate to ammonium before its assimilation are crucial differences that affect plant metabolism and growth. Ammonium promotes better root growth than nitrate, which could be used to increase the performance during the field establishment of conifer seedlings. Additionally, other differences were found in the photosynthetic organs, where nitrate induced important changes correlated with the decreased growth of pine seedlings. A differential accumulation of nitrate and ammonium occurred in the pine organs and buffered the individual effects induced by each molecule. The role of pine stems in the storage of N compounds, such as nitrate and L-asparagine, and the interaction between photosynthetic metabolism and nitrate will require further research efforts in the near future.

## Supplementary Materials

The following are available online at https://www.mdpi.com/2223-7747/9/4/481/s1, Figure S1: Heatmap of the metabolite profile data, Figure S2: L-Arginine amounts, Table S1: Metabolite profile results, Table S2: qPCR primer list.
