# Supplementary material for: Inorganic Nitrogen Form Determines Nutrient Allocation and Metabolic Responses in Maritime Pine Seedlings
Source: Plants (Basel). 2020 Apr 9;9(4):481. doi: 10.3390/plants9040481 (PMC7238028; doi:10.3390/plants9040481)
Supplement: Supplementary file 1 [file plants-09-00481-s001.zip › Table S2.docx]

**Table S2.** qPCR primer list.

| **Primer Name** | **ID [1]** | **Sequence** |
| --- | --- | --- |
| SKP1/ASK1-F | unigene18128 | ATGCTGGACAGGCTTTGAAC |
| SKP1/ASK1-R | unigene18128 | GAGTTGCTCCGAGATCTTTACA |
| SLAP-F | unigene1135 | AGTATGCTAAGGAATCGTGCCT |
| SLAP-R | unigene1135 | GTCCATAATTACACACGAACAGA |
| qNR-F | unigene1771 | ATCCCCTCCCACATCCTGTTAT |
| qNR-R | unigene1771 | ACAGAGATCAGGAGTGCTCAAT |
| qNiR-F | unigene2952 | TTCCAATTCTCATCCATCGCCA |
| qNiR-R | unigene2952 | TCGGCTAAGCTATTGGAGAAACT |
| qGS1a-F | isotig10070 | ATCGAGGAGCTTCAGTTAGAGTGG |
| qGS1a-R | isotig10070 | TGGTCGTCTCAGCAATCATAGAAGT |
| qGS1b-F | unigene10503 | CCCAATTGTTTGTGGGGGATA |
| qGS1b-R | unigene10503 | CTGAATGACAAACTAGACACTG |
| qFd_GOGAT-F | unigene9110 | ATTTGTTGCCATAGTGTGAGCG |
| qFd_GOGAT-R | unigene9110 | GGTCTCCAGGTCTAGAGGATGT |
| qNADH_GOGAT-F | isotig26980 | CATAACAAGCCACTCACATGCC |
| qNADH_GOGAT-R | isotig26980 | CTTGGACCAGGTAGTTGATGCT |
| qAlaAT1-F | unigene1013 | GTGCTTATTTTATGTCGGGCAAC |
| qAlaAT1-R | unigene1013 | GCACTACCAACCTGCAAACTAG |
| qAlaAT2-F | isotig29148 | ACCTGATGCGAGTTGTATGAAAC |
| qAlaAT2-R | isotig29148 | GCTGTTATGGGAGAGAGGGC |
| qGGT-F | isotig28624 | TGTTTGGTTTGGTTCAGGACTC |
| qGGT-R | isotig28624 | ACTATACAGCACCGCAGACATT |
| qAspAT1-F | unigene4809 | GCCTGAACAAAGATCAAGTTGCA |
| qAspAT1-R | unigene4809 | GTTTTAGAGCTCAGACCTGCCA |
| qAspAT2-F | unigene17743 | TGACTCCAGAGCAAGTTGACC |
| qAspAT2-R | unigene17743 | TGCCAAGTATTCAACATTGCCG |
| qAspAT3-F | isotig09767 | GAAGCACCTGACATCACATTGG |
| qAspAT3-R | isotig09767 | ATGAAAGATCGGTAAGCAGCCA |

1. Cañas, R. A.; Li, Z.; Pascual, M. B.; Castro-Rodríguez, V.; Ávila, C.; Sterck, L.; Van de Peer, Y.; Cánovas, F. M. The gene expression landscape of pine seedling tissues. *Plant Journal* **2017**, *91*, 1064-1087. doi: 10.1111/tpj.13617
