# Supplementary figures and images for: Inorganic Nitrogen Form Determines Nutrient Allocation and Metabolic Responses in Maritime Pine Seedlings
Source: Plants (Basel). 2020 Apr 9;9(4):481. doi: 10.3390/plants9040481 (PMC7238028; doi:10.3390/plants9040481)

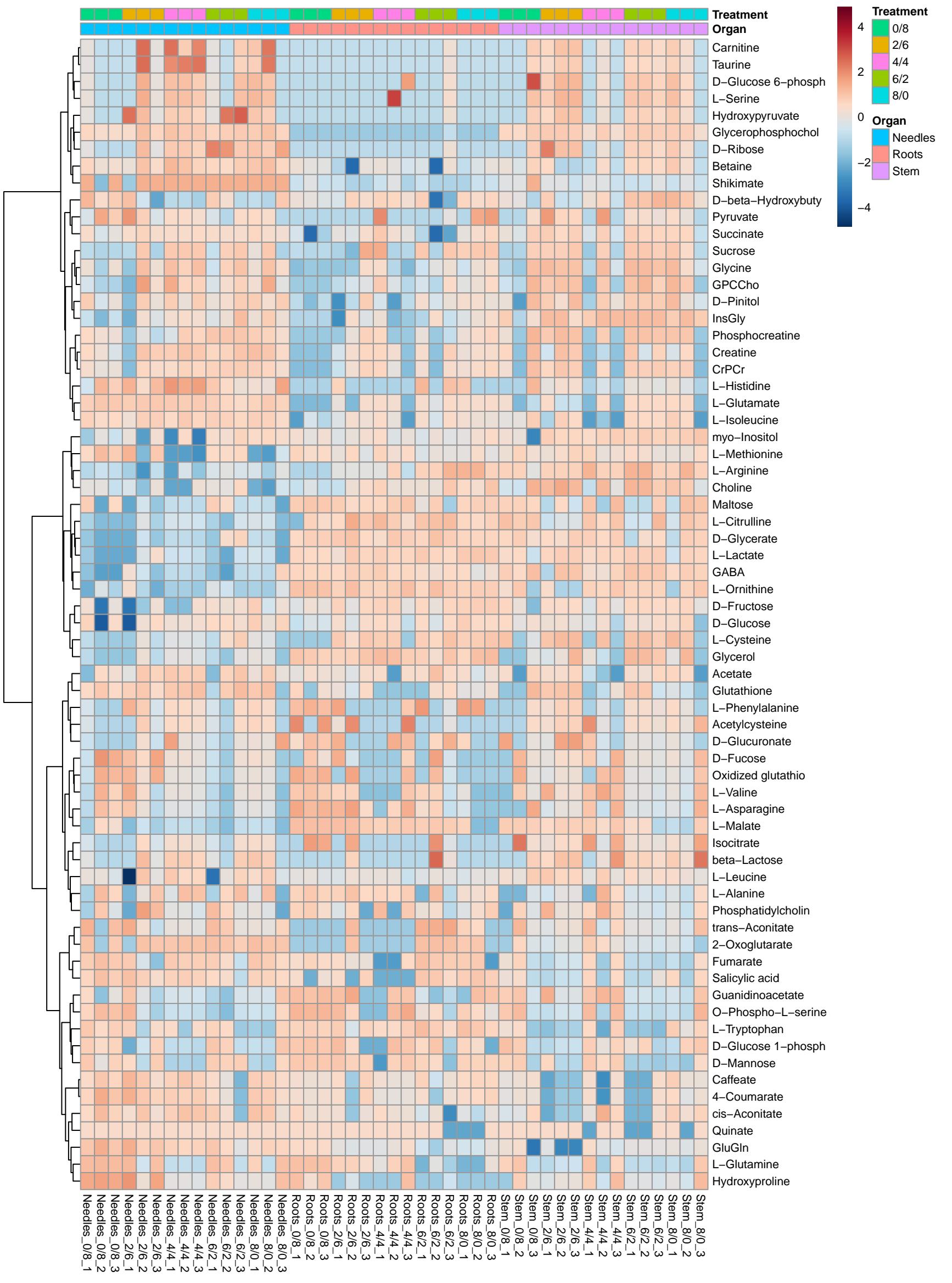

Supplement: Supplementary file 1 [file plants-09-00481-s001.zip › Figure S1.pdf]

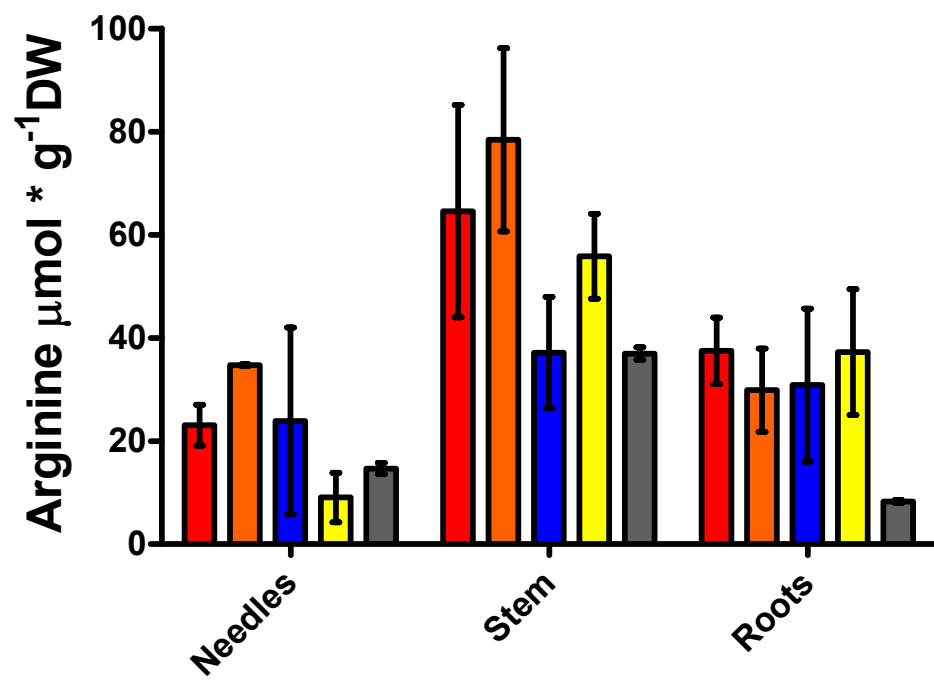

Supplement: Supplementary file 1 [file plants-09-00481-s001.zip › Figure S2.pdf]
